# Computational analysis of protein synthesis, diffusion, and binding in compartmental biochips

**DOI:** 10.1186/s12934-023-02237-5

**Published:** 2023-11-30

**Authors:** Stefanie Förste, Ohad Vonshak, Shirley S. Daube, Roy H. Bar-Ziv, Reinhard Lipowsky, Sophia Rudorf

**Affiliations:** 1 Theory and Bio-Systems, Max Planck Institute of Colloids and Interfaces, 14476 Potsdam, Germany; 2 Department of Chemical and Biological Physics, The Weizmann Institute of Science, Rehovot, 7610001 Israel; 3 Institute of Cell Biology and Biophysics, Leibniz University Hannover, 30419 Hannover, Germany

## Abstract

Protein complex assembly facilitates the combination of individual protein subunits into functional entities, and thus plays a crucial role in biology and biotechnology. Recently, we developed quasi-twodimensional, silicon-based compartmental biochips that are designed to study and administer the synthesis and assembly of protein complexes. At these biochips, individual protein subunits are synthesized from locally confined high-density DNA brushes and are captured on the chip surface by molecular traps. Here, we investigate single-gene versions of our quasi-twodimensional synthesis systems and introduce the trap-binding efficiency to characterize their performance. We show by mathematical and computational modeling how a finite trap density determines the dynamics of protein-trap binding and identify three distinct regimes of the trap-binding efficiency. We systematically study how protein-trap binding is governed by the system’s three key parameters, which are the synthesis rate, the diffusion constant and the trap-binding affinity of the expressed protein. In addition, we describe how spatially differential patterns of traps modulate the protein-trap binding dynamics. In this way, we extend the theoretical knowledge base for synthesis, diffusion, and binding in compartmental systems, which helps to achieve better control of directed molecular self-assembly required for the fabrication of nanomachines for synthetic biology applications or nanotechnological purposes.

**Supplementary Information:**

The online version contains supplementary material available at 10.1186/s12934-023-02237-5.

## Background

The plethora of tasks that biomolecules autonomously fulfill inside living cells inspired and triggered many studies on the use of peptides [1–3], nucleic acids [4–9], or polysaccharides [10–12] as building blocks for artificial nanodevices. Important properties of biomolecular nanodevices are bio-compatibility, stimuli-responsiveness, and the capability to self-assemble [13]. These devices have the potential to serve for example as delivery systems for drugs or genetic material [14, 15], for molecular computation [16], or as nanosensors [17]. However, for many applications the precise and reliable fabrication of nanomachines *via* self-assembly still remains a major challenge [18, 19]. One particular problem is the inherent complexity of the self-assembly of any multi-component nanomachine [20, 21]. Freely diffusing components can interact in many different ways, which results in unwanted side-reactions and the formation of alternative, non-functional complexes. These off-pathway reactions cause a reduction of product quality and yield by diverting and sequestering resources [22]. There are different strategies to tackle these challenges. As an example, spatio-temporal control of the assembly process is achieved by compartmentalization of the reaction chamber [23]. Furthermore, fine-tuning the stoichiometry of the individual components [24] or using molecular assembly lines [25] can reduce the probability of off-pathway reactions.

Recently, we presented systems for the in-vitro synthesis and assembly of protein complexes in quasi-twodimensional silicon chips [26]. In these systems, we separate component synthesis from component assembly by applying a soft, membrane-less compartmentalization strategy: First, we attach DNA-brushes to confined regions on the surfaces of the chips. Second, the coding sequences that are comprised by the brushes are used as templates for the synthesis of protein subunits *via* cell-free transcription and translation. Third, assembly of the subunits into protein complexes is coupled to their synthesis and occurs either in solution or by scaffolding on the surfaces of the chips, which are coated with molecular traps.

In these systems, the dynamics of the assembly of nanomachines are determined by a specific set of parameters. This set comprises the rates of component synthesis, the diffusion constants of the components, and the rate constants for component-component binding and component-trap binding. An efficient fabrication of nanomachines is only possible when these system parameters are well-adjusted to facilitate correct assembly and to suppress off-pathway reactions. Therefore, understanding how each of these parameters influences the assembly of nanomachines is a fundamental prerequisite for the effective genetic and geometric control of this process. In practice, depending on the synthesis process it might not be feasible to adjust all of the parameters or the range of possible parameter values might be highly restricted: Protein complexes assemble from defined subunits, which means that the options for molecular modifications are limited by the requirement that the functionality and integrity of the complex need to be preserved [27]. However, synthesis rates, diffusion constants and binding affinities of subunits and surface traps are in principle adjustable, e.g. *via* changing temperature, DNA-brush densities and compositions, the viscosity of the reaction solution, and the particular choice of the molecular tags for trap binding. Furthermore, the biochip allows for adjustable positioning of the DNA brushes, introducing a distinct spatial component that is unique to the system.

**Fig. 1:**
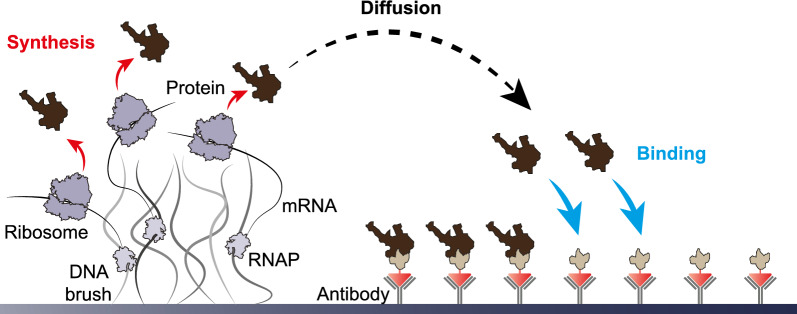
Schematic representation of a compartmentalized biochip. A DNA brush is attached to the chip surface. The encoded sequences are transcribed by RNA polymerases (light gray) and translated by ribosomes (dark gray). The synthesized proteins (brown) spread by diffusion and bind to a second type of proteins (light brown) tethered to the chip surface by antibodies

Their adjustability turns synthesis rates, diffusion constants and binding affinities into system parameters that are particularly interesting to study. In multi-component systems, the impact of the synthesis rate, the diffusion constant and the trap-binding affinity may be obscured by the complexity of the reaction network. To better understand the fundamental process, we here focus on a simplified version of the biochip studied previously [26]. In this simplified system, only one type of protein subunit is actively expressed whereas subunits of a second type are tethered to the surface of the silicon chip and serve themselves as traps for the actively expressed subunits, see Fig. 1. Consequently, assembly of two-subunit complexes can only occur at the surface but not in solution and the performance of these systems in terms of two-subunit interaction can be characterized by their trap-binding efficiency.

Here, we model these simplified biochips for protein synthesis and binding as spatially inhomogeneous reaction-diffusion systems with finite trap densities and three adjustable system parameters: (i) synthesis rate, (ii) diffusion constant, and (iii) trap-binding affinity of the expressed protein subunit. We show how the trap-binding efficiency is determined by the aforementioned system parameters and study the time evolution of protein-trap binding. In particular, we identify three distinct regimes of the trap-binding efficiency that arise from the interplay of protein synthesis and binding. Furthermore, we study the spatial distribution of bound proteins and investigate the influence of trap patterning on the trap-binding efficiency. Finally, we test the predictive power of our computational modeling by comparison with experimental data.

## Results and discussion

### Trap-binding efficiency in one-box systems with finite trap density undergoes a transition from a binding-dominated to a synthesis-dominated regime

**Fig. 2:**
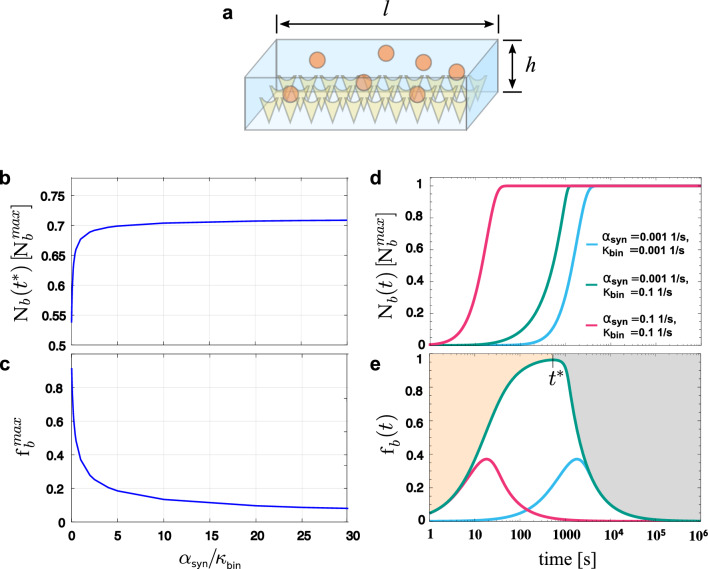
Binding dynamics of the one-box system with limited trap density. **a** One-box system with finite trap density. The quasi-twodimensional synthesis chip of height $h$ and length $l$ with $h\ll l$ is filled with a solution of proteins (orange spheres) that bind to molecular traps (yellow cones) to form protein-trap complexes. **b** Number $\rm{N}_\mathrm{b}(t^*)$ of bound proteins at $t=t^*$ and **c** maximum of the trap-binding efficiency $\rm{f}_\mathrm{b}^\mathrm{max}=\rm{f}_\mathrm{b}(t^*)$ for different ratios of synthesis rate $\alpha _\mathrm{syn}$ and binding affinity $\kappa _\mathrm{bin}$. **d** Number $\rm{N}_\mathrm{b}(t)$ of proteins bound to traps normalized to the maximal value $\rm{N}_\mathrm{b}^\mathrm{max}$ and **e** time-evolution of the trap-binding efficiency $\rm{f}_\mathrm{b}(t)$ for different choices of synthesis rate $\alpha _\mathrm{syn}$ and binding affinity $\kappa _\mathrm{bin}$. Note that the maximum of the trap-binding efficiency $\rm{f}_\mathrm{b}^\mathrm{max}$ is determined by the ratio of synthesis rate $\alpha _\mathrm{syn}$ and binding affinity $\kappa _\mathrm{bin}$, see also (**c**). For the light blue and magenta lines this ratio is one, thus $\rm{f}_\mathrm{b}^\mathrm{max}$ is identical but reached at different time points. Background color indicates binding-dominated (orange) and synthesis-dominated (gray) regime for $\alpha _\mathrm{syn}= {0.001\,\mathrm{\text {s}^{-1}}}$ and $\kappa _\mathrm{bin}= {0.1\,\mathrm{\text {s}^{-1}}}$. Transition from the former to the latter occurs at $t= t^*$, i.e., when the maximum $\rm{f}_\mathrm{b}^\mathrm{max}$ of the trap-binding efficiency is reached

We consider a quasi-twodimensional system with height $h$ and length $l$ for which $h\ll l$, i.e., a very flat box as sketched in Fig. 2a). The box is filled with a solution of proteins, where *n* new proteins are added to the box per unit of time [*t*] at the constant synthesis rate ${\alpha _{{\text{syn}}}} \equiv n/[t]$. Protein synthesis is realized by in-vitro synthesis within the solution. The bottom of the box is coated with traps to which free proteins bind in a quasi-irreversible manner with probability *p* per unit of time [*t*], i.e., with binding affinity ${\kappa _{{\text{bin}}}} \equiv p/[t]$. The trap density is finite and each trap can only bind one protein. Therefore, the number ${{\text{N}}_{\text{b}}}({\text{t}})$ of bound proteins is always less or equal than a maximal value ${\text{N}}_{\text{b}}^{{\text{max}}}$, which corresponds to the total number of traps provided the lateral size of the proteins is smaller than the trap separation. We assume that the solution is perfectly mixed at all times so that the time evolution of the numbers ${{\text{N}}_{\text{u}}}({\text{t}})$ and ${{\text{N}}_{\text{b}}}({\text{t}})$ of unbound and bound proteins is described by

1 $$\frac{{\text{d}}}{{{\text{d}}t}}{{\text{N}}_{\text{u}}}({\text{t}}){\text{ =  - }}{\kappa _{{\text{bin}}}}{\mkern 1mu} {{\text{N}}_{\text{u}}}({\text{t}})\left( {{\text{N}}_{\text{b}}^{\max }{\text{ - }}{{\text{N}}_{\text{b}}}({\text{t}})} \right){\text{ + }}{\alpha _{{\text{syn}}}}{\text{,}}$$

2 $$\frac{\hbox {d}}{\hbox {d}t}\rm{N}_\mathrm{b}(t)= \kappa _\mathrm{bin}\,\rm{N}_\mathrm{u}(t) \left( \rm{N}_\mathrm{b}^\mathrm{max} - \rm{N}_\mathrm{b}(t) \right) \,,$$

and mass conservation

3 $$\rm{N}(t)\equiv \rm{N}_\mathrm{b}(t) + \rm{N}_\mathrm{u}(t) = \alpha _\mathrm{syn}t + \rm{N}_\mathrm{b}(0) + \rm{N}_\mathrm{u}(0)$$

with initial numbers $\rm{N}_\mathrm{b}(0)$ and $\rm{N}_\mathrm{u}(0)$ of bound and unbound proteins. A solution of this initial value problem is given by Eq. (15) in the Methods section. In the following, we investigate the time evolution of the fraction $\rm{f}_\mathrm{b}(t)$ of bound proteins in the box

4 $$\begin{aligned} \rm{f}_\mathrm{b}(t)&\equiv \rm{N}_\mathrm{b}(t)/\rm{N}(t)\,&\end{aligned}$$

and refer to $\rm{f}_\mathrm{b}(t)$ as the *trap-binding efficiency* of the system. For more efficient systems, the maximum $\rm{f}_\mathrm{b}^\mathrm{max}$ of the trap-binding efficiency gets closer to the highest possible value 1, is reached at an earlier point in time $t=t^*$ after protein synthesis has started and stays at a high level for a longer time than for less efficient systems. Figure 2 shows examples for the number $\rm{N}_\mathrm{b}(t^*)$ of bound proteins at $t=t^*$ (Fig. 2b), the maximum $\rm{f}_\mathrm{b}^\mathrm{max}=\rm{f}_\mathrm{b}(t^*)$ of the trap-binding efficiency (Fig. 2c), the time evolution of the number $\rm{N}_\mathrm{b}(t)$ of bound proteins (Fig. 2d), and the time evolution of the trap-binding efficiency $\rm{f}_\mathrm{b}(t)$ (Fig. 2e) for different synthesis rates $\alpha _\mathrm{syn}$ and binding affinities $\kappa _\mathrm{bin}$: As expected, the number $\rm{N}_\mathrm{b}(t)$ of bound proteins increases and approaches the plateau value $\rm{N}_\mathrm{b}^\mathrm{max}$. The trap-binding efficiency $\rm{f}_\mathrm{b}(t)$ increases after the onset of protein synthesis at a rate that is determined by the binding affinity $\kappa _\mathrm{bin}$. For longer timescales, the synthesis rate $\alpha _\mathrm{syn}$ determines how fast $\rm{f}_\mathrm{b}(t)$ approaches zero as the long-term behavior of the trap-binding efficiency is governed by $\rm{f}_\mathrm{b}(t) \propto \rm{N}_\mathrm{b}^\mathrm{max}\left( \alpha _\mathrm{syn}t \right) ^{-1}$. Thus, for each particular choice of $\kappa _\mathrm{bin}$ and $\alpha _\mathrm{syn}$, we can distinguish a binding-dominated and a synthesis-dominated regime for the time evolution of the trap-binding efficiency. The transition from the first to the latter regime occurs at time $t^*$ when the trap-binding efficiency has reached its maximum $\rm{f}_\mathrm{b}^\mathrm{max}$ and starts to decrease, see Fig. 2e). At this time, many traps are already saturated, whereas the total number of proteins keeps to increase linearly. The time $t^*$, the trap-binding efficiency maximum $\rm{f}_\mathrm{b}^\mathrm{max}$ and the number $\rm{N}_\mathrm{b}(t^*)$ of bound proteins at $t = t^*$ depend on the binding affinity $\kappa _\mathrm{bin}$ and the synthesis rate $\alpha _\mathrm{syn}$, see Figs. 2 b), c), and e). They can be determined by solving the initial value problem defined in Eqs. (1) and (2) together with $\frac{\hbox {d}}{\hbox {d}t} \rm{f}_\mathrm{b}(t)|_{t = t^*} = 0$.

### Trap-binding efficiency in two-box systems with inhomogeneous synthesis and slow diffusion exhibits three distinct regimes

**Fig. 3:**
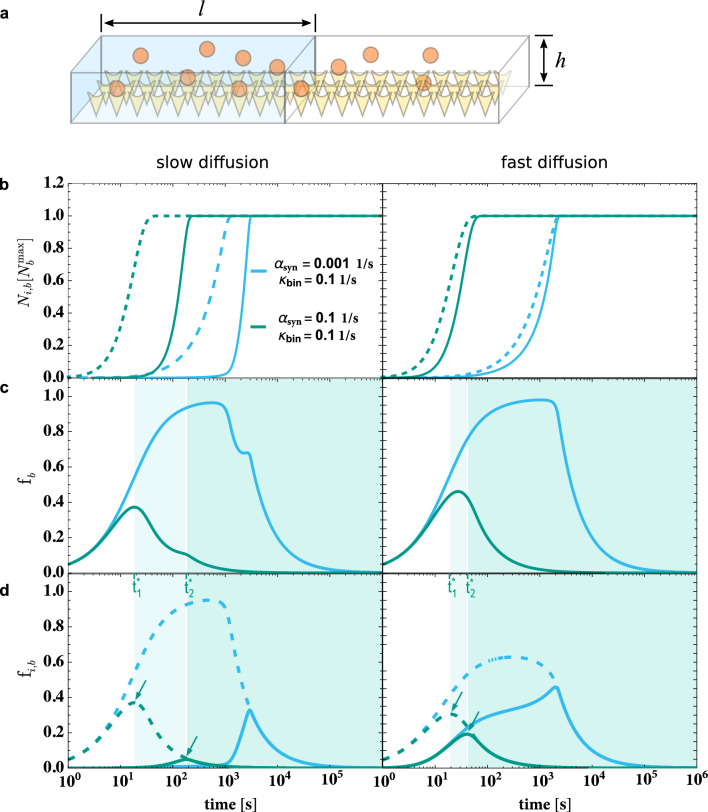
Binding dynamics in a two-box system with diffusion rates $\widetilde{D}={0.001\,\mathrm{\text {s}^{-1}}}$ (left) and $\widetilde{D}={0.1\,\mathrm{\text {s}^{-1}}}$ (right), and synthesis rates $\alpha _\mathrm{syn}$ and binding affinities $\kappa _\mathrm{bin}$ as indicated. **a** The two-box system with box height $h$, box length $l$, and $h<< 2\,l$. Proteins (orange spheres) can freely diffuse between both boxes and bind to traps (yellow cones). New proteins are synthesized only in the left box (blue). **b** Numbers $\rm{N}_{i,\rm{b}}(t)$ of bound proteins in box $i=1$ (dashed lines) and $i=2$ (solid lines). Because synthesis is restricted to box 1, saturation of traps in box 2 is lagging behind. This lag is not observable in well-mixed systems with high diffusion rates. **c** In two-box systems, the trap-binding efficiency $\rm{f}_\mathrm{b}(t)$ exhibits a pure binding-dominated ($0 \le t < t^*_\mathrm{1}$), a mixed ($t^*_\mathrm{1} \le t < t^*_\mathrm{2}$), and a pure synthesis-dominated regime ($t \ge t^*_\mathrm{2}$) as indicated by background shading. d) Individual trap binding efficiencies $\rm{f}_\mathrm{i,b}(t)\equiv \rm{N}_{i,\rm{b}}(t)/\rm{N}_i(t)$ of box $i=1$ (dashed lines) and $i=2$ (solid lines). Transitions from the binding-dominated to the synthesis-dominated regimes in the boxes 1 and 2 occur at time points $t^*_\mathrm{1}$ and $t^*_\mathrm{2}$, respectively. Arrows point to the maxima of $\rm{f}_\mathrm{1,b}(t)$ and $\rm{f}_\mathrm{2,b}(t)$ at $t = t^*_\mathrm{1}$ and $t = t^*_\mathrm{2}$, respectively

We extend the one-box system introduced above by a second box that is identical and directly connected to the first one, see Fig. 3a). As in the one-box system, both boxes 1 and 2 of the two-box system are filled with a protein solution and their bottom is coated with traps that the proteins can bind to. Both boxes of the system are open in the sense that proteins can freely diffuse between them with *diffusion rate* $\widetilde{D}$. The diffusion rate $\widetilde{D}$ is defined as the diffusion constant $D$ of the proteins scaled by the box length $l$, $\widetilde{D}\equiv D/l^2$, and is expressed by the inverse unit of time $[t]^{-1}$. In contrast to the one-box system, protein synthesis in the two-box system is not homogeneous. Instead, proteins are only synthesized in box 1 but not in box 2. Thus, the dynamics of the numbers $\rm{N}_{i,\rm{u}}(t)$ and $\rm{N}_{i,\rm{b}}(t)$ of unbound and bound proteins in boxes $i=1$ and 2 are described by

5 $$\begin{aligned} \frac{\hbox {d}}{\hbox {d}t}&= \widetilde{D}\left( \rm{N}_{2,\rm{u}}(t) - \rm{N}_{1,\rm{u}}(t) \right) - \kappa _\mathrm{bin}\rm{N}_{1,\rm{u}}(t) \left( \rm{N}_\mathrm{1,b}^\mathrm{max}-\rm{N}_{1,\rm{b}}(t) \right) + \alpha _\mathrm{syn}\,,&\end{aligned}$$

6 $$\begin{aligned} \frac{\hbox {d}}{\hbox {d}t}&= \kappa _\mathrm{bin}\rm{N}_{1,\rm{u}}(t) \left( \rm{N}_\mathrm{1,b}^\mathrm{max}-\rm{N}_{1,\rm{b}}(t) \right) \,,&\end{aligned}$$

7 $$\begin{aligned} \frac{\hbox {d}}{\hbox {d}t}&= \widetilde{D}\left( \rm{N}_{1,\rm{u}}(t) - \rm{N}_{2,\rm{u}}(t) \right) - \kappa _\mathrm{bin}\rm{N}_{2,\rm{u}}(t) \left( \rm{N}_\mathrm{2,b}^\mathrm{max}-\rm{N}_{2,\rm{b}}(t) \right) \,, \quad \text {and}&\end{aligned}$$

8 $$\begin{aligned} \frac{\hbox {d}}{\hbox {d}t}&= \kappa _\mathrm{bin}\rm{N}_{2,\rm{u}}(t) \left( \rm{N}_\mathrm{2,b}^\mathrm{max}-\rm{N}_{2,\rm{b}}(t) \right) ,&\end{aligned}$$

with synthesis rate $\alpha _\mathrm{syn}$ and binding affinity $\kappa _\mathrm{bin}$ defined in the context of Eq. (1). In addition, mass conservation leads to the relationship

9 $$\begin{aligned} \rm{N}(t)& = \rm{N}_{1,\rm{u}}(t) + \rm{N}_{1,\rm{b}}(t) + \rm{N}_{2,\rm{u}}(t) + \rm{N}_{2,\rm{b}}(t) \\ &= \alpha _\mathrm{syn}t + \rm{N}_{1,\rm{u}}(0) + \rm{N}_{1,\rm{b}}(0) + \rm{N}_{2,\rm{u}}(0) + \rm{N}_{2,\rm{b}}(0)\,. \end{aligned}$$

The time evolution of the numbers of bound proteins in box 1 and 2 is shown in Fig. 3b): Due to the spatially inhomogeneous synthesis of proteins in the two-box system, the traps in box 2 are saturated at a later point in time than the traps in box 1. For a given synthesis rate $\alpha _\mathrm{syn}$ and binding affinity $\kappa _\mathrm{bin}$, the lag time depends on the diffusion rate $\widetilde{D}$ and is negligible for very fast diffusion (i.e., in well-mixed systems) as expected. A diffusion that is slow enough to cause a lag in the saturation of traps in box 2 has also an impact on the trap-binding efficiency $\rm{f}_\mathrm{b}(t)\equiv \left( \rm{N}_{1,\rm{b}}(t) + \rm{N}_{2,\rm{b}}(t)\right) /\rm{N}(t)$ of the two-box system. The time evolution of $\rm{f}_\mathrm{b}(t)$ can be now divided into three regimes, see Fig. 3c): At first, the system is in a *pure binding-dominated regime* (with boxes 1 and 2 being both in the binding-dominated regime). In this regime, the trap-binding efficiency $\rm{f}_\mathrm{b}(t)$ monotonically increases. At $t = t^*_\mathrm{1}$ box 1 undergoes a transition from the binding-dominated to the synthesis-dominated regime, see Fig. 3d). At this point in time, the two-box system enters a *mixed regime*, because box 2 is still in the binding-dominated regime. The system remains in the mixed regime until $t = t^*_\mathrm{2}$ when box 2 also undergoes a transition to the synthesis-dominated regime and the two-box system enters the *pure synthesis-dominated regime*. For fast diffusion, the mixed regime is short-lived because the lag time $t^*_\mathrm{2} - t^*_\mathrm{1}$ becomes small. Note that the time evolution of the trap-binding efficiency in the mixed regime strongly varies depending on the system parameters. In contrast, in the pure synthesis-dominated regime the trap-binding efficiency always monotonically decreases and asymptotically approaches $\rm{f}_\mathrm{b}(t) \propto \left( \rm{N}_\mathrm{1,b}^\mathrm{max}+\rm{N}_\mathrm{2,b}^\mathrm{max}\right) \left( \alpha _\mathrm{syn}t \right) ^{-1}$.

### Binding and diffusion in quasi-realistic systems with finite trap densities and inhomogeneous synthesis

To study diffusion and binding for finite trap densities in a more realistic system, we investigate a system comprised of $M\gg 1$ boxes. The number $\rm{N}_i(t)$ of proteins in box $i=1,...,M$ changes over time due to the synthesis of new proteins and their diffusion between neighboring boxes. The proteins freely diffuse with diffusion rate $\widetilde{D}$ until they bind to traps, which coat the bottom of the boxes, with binding affinity $\kappa _\mathrm{bin}$. Binding can only occur as long as free traps are available, which implies that the number $\rm{N}_{i,\rm{b}}(t)$ of bound proteins in box *i* cannot exceed its maximum $\rm{N}_\mathrm{i,b}^\mathrm{max}$. Like in the two-box system introduced above, the synthesis of new proteins can be restricted to a specific subset of boxes. We here focus on a system with $M=100$ boxes and - corresponding to the experimental system - boxes $i=i_\mathrm{syn}=12,...,19$ serving as sources for new proteins of which each provides new proteins at a synthesis rate $\alpha _\mathrm{syn}$. As an example, the 100-box system could be a spatially discretized model of a quasi-twodimensional biochip of length $L= 1\,\hbox {mm}$, where each box represents a section with length $l=10\upmu \hbox {m}$ of the chip. Note that in this case the box index *i* indicates the discretized position relative to the biochip and $L=l\cdot M$.

Equivalently to the two-box system described above, the time-evolution of the numbers $\rm{N}_{i,\rm{u}}(t)$ and $\rm{N}_{i,\rm{b}}(t)$ of free and bound proteins in box *i* is given by

10 $$\begin{aligned} \frac{\hbox {d}}{\hbox {d}t}&= \kappa _\mathrm{bin}\rm{N}_{i,\rm{u}}(t) \left( \rm{N}_\mathrm{i,b}^\mathrm{max}-\rm{N}_{i,\rm{b}}(t) \right) \quad \text {for i=1,\,...,M}\,,&\end{aligned}$$

11 $$\begin{aligned} \frac{\hbox {d}}{\hbox {d}t}&= \widetilde{D}\left( \rm{N}_{i+1,\rm{u}}(t) + \rm{N}_{i-1,\rm{u}}(t) - 2\,\rm{N}_{i,\rm{u}}(t) \right) \\ &\quad- \kappa _\mathrm{bin}\rm{N}_{i,\rm{u}}(t) \left( \rm{N}_\mathrm{i,b}^\mathrm{max}-\rm{N}_{i,\rm{b}}(t) \right) + \alpha _\mathrm{syn}\left[ i=i_\mathrm{syn} \right] \end{aligned}$$

for $$i=2,...,M-1$$, and

12 $$\begin{aligned} \frac{\hbox {d}}{\hbox {d}t}&= \widetilde{D}\left( \rm{N}_{2,\rm{u}}(t) - \rm{N}_{1,\rm{u}}(t) \right) \\ &\quad- \kappa _\mathrm{bin}\rm{N}_{1,\rm{u}}(t) \left( \rm{N}_\mathrm{1,b}^\mathrm{max}-\rm{N}_{1,\rm{b}}(t) \right) \\ &\quad+ \alpha _\mathrm{syn}\left[ 1=i_\mathrm{syn} \right] \,,&\end{aligned}$$

13 $$\begin{aligned} \frac{\hbox {d}}{\hbox {d}t}&= \widetilde{D}\left( \rm{N}_{M-1,\rm{u}}(t) - \rm{N}_{M,\rm{u}}(t) \right) \\ &\quad- \kappa _\mathrm{bin}\rm{N}_{M,\rm{u}}(t) \left( \rm{N}_\mathrm{M,b}^\mathrm{max}-\rm{N}_{M,\rm{b}}(t) \right)\\ &\quad + \alpha _\mathrm{syn}\left[ M=i_\mathrm{syn} \right] \,,&\end{aligned}$$

where the Iverson bracket $\left[ i=i_\mathrm{syn} \right] = 1$ if box *i* is a source for new proteins and 0 otherwise. In addition, mass conservation must hold, which implies

14 $$\begin{aligned} \sum _{i=1}^M\left( \rm{N}_{i,\rm{u}}(t) + \rm{N}_{i,\rm{b}}(t)\right) = \sum _{i=1}^M\left( \rm{N}_{i,\rm{u}}(0) + \rm{N}_{i,\rm{b}}(0) + \alpha _\mathrm{syn}t \left[ i=i_\mathrm{syn} \right] \right) \,. \end{aligned}$$

We solved the system of equations (10) to (14) numerically by using Euler’s method with a fixed discrete time step.

### Trap-binding efficiency in quasi-realistic systems

**Fig. 4:**
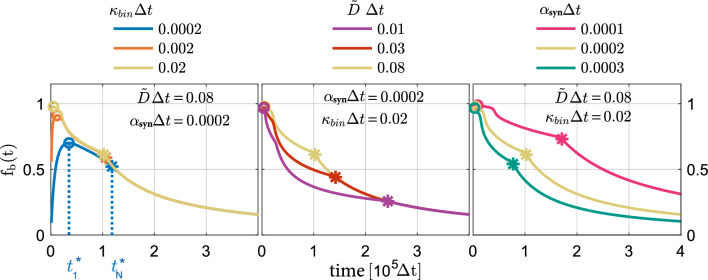
Trap-binding efficiency $\rm{f}_\mathrm{b}(t)$ in 100-box systems with synthesis rate $\alpha _\mathrm{syn}$, binding affinity $\kappa _\mathrm{bin}$ and diffusion rate $\widetilde{D}$. Time-evolution of the trap-binding efficiency $\rm{f}_\mathrm{b}(t)$ for a 100-box system with synthesis of proteins restricted to boxes 12 to 19 and system parameters as indicated. Circles and stars mark the transition from the pure binding-dominated to the mixed regime at $t^*_{1}$ and from the mixed to the pure synthesis-dominated regime at $t^*_{100}$, respectively

At first, we study the time-dependent trap-binding efficiency $\rm{f}_\mathrm{b}(t)$ of the 100-box system, which is defined equivalently to Eq. (4) by the ratio of the total number $\rm{N}_\mathrm{b}(t)\equiv \sum _{i=1}^{100} \rm{N}_{i,\rm{b}}(t)$ of bound proteins and the total number $\rm{N}(t) \equiv \sum _{i=1}^{100}\rm{N}_i(t)$ of all proteins in the system. Like in the simple two-box system described above, the time evolution $\rm{f}_\mathrm{b}(t)$ of the trap-binding efficiency in the 100-box system can also be divided into three distinct regimes, see Fig. 4: (i) a pure binding-dominated regime, where all boxes are in the binding-dominated regime; (ii) a mixed regime with at least one box in the binding-dominated regime and at least one box in the synthesis-dominated regime; and (iii) a pure synthesis-dominated regime, where all boxes are in the synthesis-dominated regime. In all three regimes, the trap-binding efficiency is higher in systems with a faster diffusion, a lower synthesis of new proteins, or a higher trap binding affinity. A lower synthesis of new proteins, a faster diffusion, or a smaller binding affinity of proteins and traps attenuate the accumulation of trapped proteins in the proximity of the synthesis sites and, thus, cause a delay of the transition from the pure binding-dominated to the mixed regime. A transition from the mixed to the pure synthesis-dominated regime occurs when the box that is the most distant from the synthesis sites enters the synthesis-dominated regime. Therefore, this transition is delayed by slower diffusion, weaker binding, and lower synthesis.

### Inhomogeneities in the trap density modulate the distribution of bound molecules

**Fig. 5:**
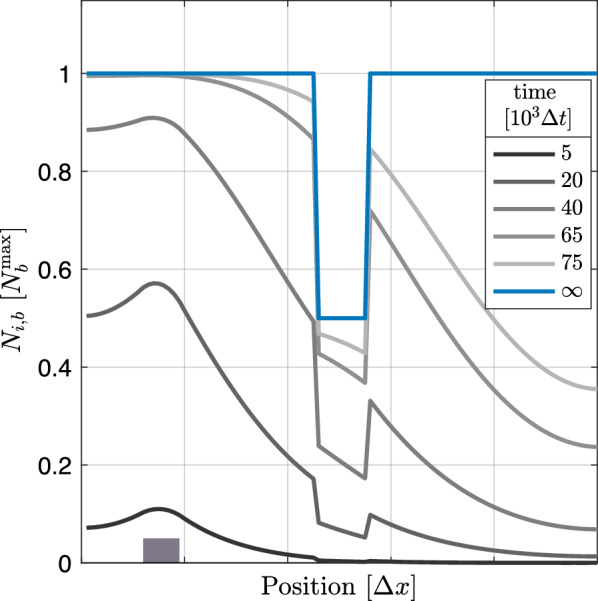
Inhomogeneities in the trap pattern of a 100-box system can be reflected by the distributions of bound proteins long before the traps get saturated. Local numbers of bound proteins $\rm{N}_{i,\rm{b}}(t)$ for binding affinity $\kappa _\mathrm{bin}={2e-4\,.} \Delta t^{-1}$, diffusion rate $\widetilde{D}={8.3e-2\,.} \Delta t^{-1}$, synthesis rate $\alpha _\mathrm{syn}={2e-4\,.} \Delta t^{-1}$, and specific points in time as indicated in the figure legend. Numbers are normalized to the overall largest value of the local maximal numbers $\rm{N}_\mathrm{i,b}^\mathrm{max}$ of bound proteins, i.e., $\rm{N}_\mathrm{b}^\mathrm{max}=\max (\rm{N}_\mathrm{i,b}^\mathrm{max}: i=1,...,100)$. The local maximal numbers $\rm{N}_\mathrm{i,b}^\mathrm{max}$ are equivalent to the local numbers of traps (solid blue line, “$\infty$”), i.e., the inhomogeneous trap pattern. The gray box indicates the area of protein synthesis

So far, we discussed systems with a homogeneous trap density, i.e, with a location-independent maximal number of bound proteins per box $\rm{N}_\mathrm{i,b}^\mathrm{max}=\rm{N}_\mathrm{b}^\mathrm{max}$ for all boxes $i=1,...,M$. In this paragraph, we investigate how inhomogeneities in the trap density modulate the dynamics and the localization of trap binding. In an experimental setup, these inhomogeneities may arise from an intended trap patterning, an incomplete coating of the reaction chamber or microfluidic chip with traps, or from impurities in the reaction solution that block the traps in an unspecific manner before proteins can bind. In Fig. 5 we plotted distributions of bound proteins at specific times for binding affinity $\kappa _\mathrm{bin}={2e-4\,.} \Delta t^{-1}$, diffusion rate $\widetilde{D}={8.3e-2\,.} \Delta t^{-1}$, synthesis rate $\alpha _\mathrm{syn}={2e-4\,.} \Delta t^{-1}$ and the trap pattern indicated by the blue solid line. The distribution of bound proteins may reflect the trap pattern already at early times when most of the traps are still unoccupied because the probability that a protein gets trapped is reduced in areas with a lower trap density. How clearly the trap pattern is imprinted in the distribution of bound proteins at a given point in time can differ for binding reaction-limited systems (with relatively high diffusion constants and low binding affinities) and diffusion-limited systems (with relatively low diffusion constants and high binding affinities). For the trap pattern in Fig. 5, the distributions of bound proteins reflect the inhomogeneous trap density already at early time points if the system is binding reaction-limited and not diffusion-limited.

### Systems with spatially restricted binding domains

**Fig. 6:**
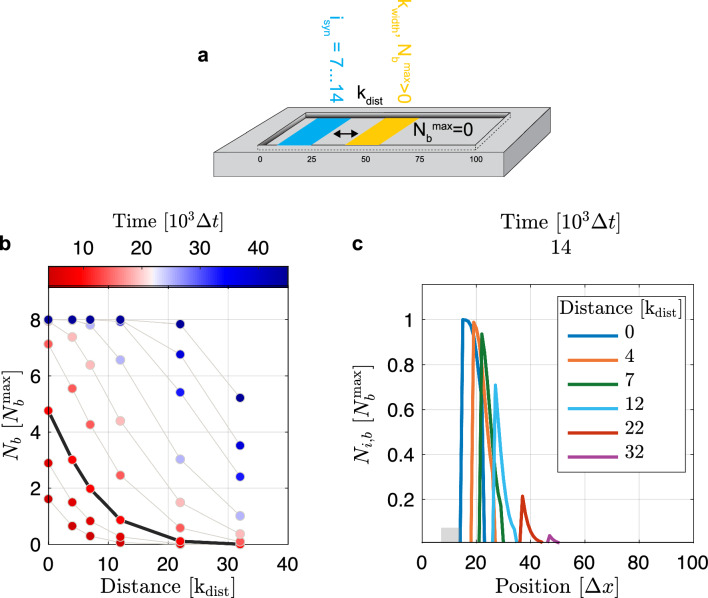
Binding in spatially restricted domains. **a** The system consists of 100 boxes where binding is restricted to 8 consecutive boxes $i_\mathrm{trap}$ such that $\rm{N}_\mathrm{i,b}^\mathrm{max} = \rm{N}_\mathrm{b}^\mathrm{max}$ for $i=i_\mathrm{trap}$ and $\rm{N}_\mathrm{i,b}^\mathrm{max} =0$ elsewhere. Synthesis sites are at boxes $i_\mathrm{syn} = 7,\,...,14$ (blue) and boxes with traps (yellow) are located in a specific distance $k_\mathrm{dist}$ to the synthesis site as indicated. Further system parameters are binding affinity $\kappa _\mathrm{bin}={2e-2\,.} \Delta t^{-1}$, diffusion rate $\widetilde{D}={9.8e-3\,.} \Delta t^{-1}$, and synthesis rate $\alpha _\mathrm{syn}={2e-4\,.} \Delta t^{-1}$. **b** Total number of bound proteins in the biochip at different time points and distances between synthesis sites and traps. The bold line corresponds to the time point in (**c**), which shows the distributions of bound proteins for different trap distances $k_\mathrm{dist}$

For certain applications or for technical reasons, binding of proteins to traps might be restricted to a specific region within the chip surface. We model such systems by setting the maximal number of bound proteins $\rm{N}_\mathrm{i,b}^\mathrm{max} = 0$ for all boxes *i* except for $i=i_\mathrm{trap}=15+k_\mathrm{dist},...,15+k_\mathrm{dist}+k_\mathrm{width}$, where $k_\mathrm{dist}$ indicates the distance between the synthesis sites and the trap region, and $k_\mathrm{width}$ refers to the number of consecutive boxes that define the trap region. We set $k_\mathrm{width} = 8$ and $\rm{N}_\mathrm {i_\mathrm{trap},b}^\mathrm{max} = \rm{N}_\mathrm{b}^\mathrm{max} > 0$. Figure 6 shows total numbers and exemplary distributions of bound proteins at several points in time and for different trap positions. Proteins that diffuse faster and are less easily captured by traps have a greater chance to spread throughout the whole reaction chamber such that the number of proteins in solution gets more homogeneous. Thus, in these systems the total number of bound proteins as a function of time depends much less on the distance $k_\mathrm{dist}$ between synthesis sites and traps compared to systems with slowly diffusing proteins with a high trap-binding affinity. For a system with known synthesis rate and diffusion rate, these differences in the trap-position dependence could be exploited to determine an unknown binding affinity $\kappa _\mathrm{bin}$: Using the binding affinity as the fit parameter, the computed total number of bound proteins could be fitted to the measured amount of bound proteins, either at a specific time point for different trap positions or at several time points for a specific trap position.

### Experimental validation: protein synthesis, diffusion, and trap binding in a quasi-twodimensional silicon-based compartmental system

In this section, we compare experimental data to predictions from our computational 100-box system to validate our modeling approach. The experimental setup has been described previously [26]. Briefly, we used compartments of dimensions $200\,{\upmu }\hbox {m}$ width, $1000\,{\upmu }\hbox {m}$ length, and $2\,{\upmu }\hbox {m}$ height. First, we uniformly immobilized DNA along the width of the compartment, close to one edge. Here, we used DNA encoding the bacteriophage protein gp10 as a model protein. Antibody-based traps (tagged protein gp11-HA) were then immobilized all along the compartments, excluding the area of the DNA. After washing all non-immobilized bio-molecules, we filled the compartments with a cell-free reaction solution and sealed them. Incubating the compartments for 2–3 h enabled the synthesis, diffusion, and trapping of gp10 from the immobilized DNA to the traps to occur.

**Fig. 7:**
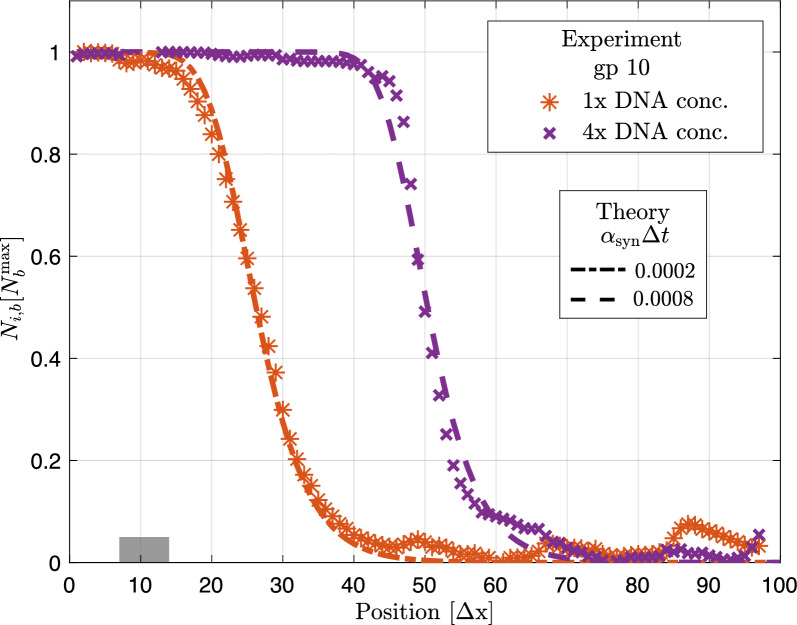
Experimental validation of the 100-box model. Fluorescence signals of trap-bound gp10 synthesized in a quasi-twodimensional, silicon-based expression system recently introduced [26] (symbols) and corresponding simulation results at $t = 21\cdot 10^3\Delta t$ (dashed lines). In the experiment, the gp10 synthesis rate is roughly proportional to the amount of gp10-coding DNA sequences. Simulations were performed in the 100-box system as described in the main text with synthesis sites being located at boxes $i_\mathrm{syn} = 7,...,14$, binding affinity $\kappa _\mathrm{bin}={2e-3\,.} \Delta t^{-1}$, diffusion rate $\widetilde{D}={0.045\,.}\Delta t^{-1}$ and synthesis rate as indicated. Increasing the amount of gp10-coding DNA sequences on the biochip by a factor of 4 corresponds to an increase in the simulation parameter $\alpha _\mathrm{syn}$ by roughly the same fold change

First, we studied the distribution of trapped gp10 for different fractions of coding sequences in the DNA brush. The protein synthesis rate can be controlled by the amount of coding DNA provided that all components required for protein synthesis are added in excess. Figure 7 shows that for a higher synthesis rate indeed a larger part of the chip is covered with gp10 120 min after protein synthesis has started and that our computational 100-box model reproduces the experimental data.

**Fig. 8:**
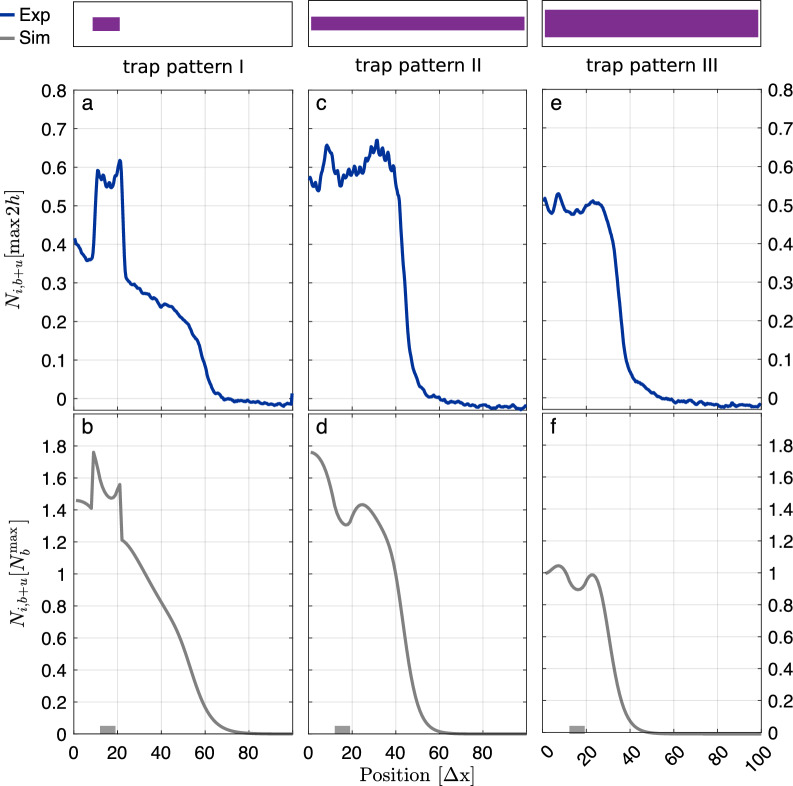
Qualitative comparison of experimental results and simulations for different trap patterns. **a**, **c**, **e** Fluorescence signals of bound and unbound ($b+u$) GFP synthesized in a quasi-twodimensional, silicon-based expression system recently introduced [26] for different trap patterns (purple bars) 1 h after start of synthesis. Signal normalized to the respective maximum value measured after 2 h. **b**, **d**, **f** Simulations of GFP synthesis, diffusion and binding in a 100-box system with synthesis sites being located at boxes $i_\mathrm{syn} = 12,\,...,19$ (gray boxes), trap patterns as in **a**, **c** and **e**, respectively, and at simulation time $t = 39\cdot 10^3\Delta t$. Number of bound and unbound GFP molecules is normalized to the respective maximal number of traps per box

Second, we checked whether the 100-box model correctly predicts the influence of trap patterns on the distribution of proteins within the chip and their binding to the traps. Instead of gp10, which requires post-staining for visualization [26], we used GFP as a model protein and reporter. GFP is a fluorescent protein and can therefore be monitored both as a freely diffusing molecule and when bound to a trap. We performed experiments with three different trap distributions, see Fig. 8: for the first trap distribution, traps were only present in proximity to the DNA brushes; for the second, traps were distributed homogeneously along the chip; for the third, the area covered by traps and, thus, the total number of traps was approximately doubled compared to the second. After start of synthesis, GFP fills both the traps and the reaction solution of the chip. The trap pattern has an impact on the distribution of GFP because the traps effectively slow down the spread of GFP within the chip, see Fig. 8. The strong correlation between simulation and experimental results suggests that the computational model provides an adequate description of protein synthesis, diffusion, and binding in compartmentalized systems introduced in [26].

## Conclusion

Recently, we introduced quasi-twodimensional, silicon-based systems for the in-vitro synthesis and assembly of bionanomachines consisting of multiple protein subunits, where we introduced a model to examine different modes of protein assembly [26]. Here, we discussed a simplified version of these systems, in which a single protein subunit is synthesized either everywhere or in a spatially defined region of the chip and is later bound to the chip surface by molecular traps. The dynamics of these simplified systems are governed by the synthesis rate $\alpha _\mathrm{syn}$, the diffusion constant $D$ of the protein, and the trap-binding affinity $\kappa _\mathrm{bin}$. We studied the influence of these parameters on the system’s trap-binding efficiency $\rm{f}_\mathrm{b}(t)$, which is defined as the time-dependent fraction of proteins that are bound to traps, see Eq. (4). In particular, we took into account that the trap density is limited. We found that systems with spatially homogeneous synthesis of proteins undergo a transition from a binding-dominated to a synthesis-dominated regime after protein synthesis has started: The first regime is characterized by a high fraction of proteins that get bound to traps, whereas in the second regime a high fraction of traps is occupied and the systems fills up with unbound proteins. Thus, the trap-binding efficiency increases in the binding-dominated regime and decreases in the synthesis-dominated regime. A spatial confinement of protein synthesis introduces more complexity to the binding dynamics: Systems with a spatially inhomogeneous synthesis that are not well-mixed also exhibit a pure binding-dominated and a pure synthesis-dominated regime, but these are separated by a mixed regime. In the mixed regime, local binding-dominated coexist with local synthesis-dominated regimes. Spatial inhomogeneities of protein synthesis are not the only way to modulate the dynamics of protein spreading and trap binding in the biochip. Further complexities can be introduced by using non-uniform distributions of traps on the surface of the synthesis systems. Depending on the system parameters, the spatial distribution of bound proteins is shaped by the trap pattern long before all traps get saturated. Therefore, designing non-uniform trap distributions provides a possibility to control both the temporal and the spatial dependence of protein-trap binding. Furthermore, we studied systems with spatially restricted binding where traps are only present in a distinct, narrow region on the surface. We showed that the system parameters determine the impact of the distance between synthesis and trap sites on the protein-trap binding dynamics. This parameter-dependence could potentially open up a new path to infer for example unknown binding affinities from systems with known synthesis rates and diffusion constants: To this end, the model presented in this work might be fitted to the fluorescence time courses of trapped proteins measured in a series of experiments with increasing distances between synthesis and trap sites. Finally, we performed experiments to validate our modeling approach. We used single-gene versions of our silicon-based compartmental system introduced in [26] to synthesize the proteins gp10 and GFP for different synthesis rates (gp10) and trap patterns (GFP). Simulations based on the here defined diffusion system with limited trap density reproduce the experimental findings, which indicates that the presented model is an adequate description of protein synthesis, diffusion and binding in quasi-twodimensional biochips. Overall, our work broadens the theoretical understanding of synthesis, diffusion, and binding processes in compartmental systems. This advancement enables improved control over directed molecular self-assembly, facilitating the fabrication of nanomachines for applications in synthetic biology or nanotechnology (Additional file 1, 2).

## Methods

### Time-dependent number of bound proteins in the one-box system

We solved the differential Eqs. (1) and (2) using the software Wolfram Mathematica. The number $\rm{N}_\mathrm{b}(t)$ of bound proteins relative to its maximal value $\rm{N}_\mathrm{b}^\mathrm{max}$ is given by

15 $$ \frac{\rm{N}_\mathrm{b}(t)}{\rm{N}_\mathrm{b}^\mathrm{max}}= 1 - {\eta }\frac{e^{- \lambda ^2 C_{\zeta }\left( t \right) ^2}}{e^{-\lambda ^2 C_{\zeta }\left( 0 \right) ^2} + \rm{N}_\mathrm{b}^\mathrm{max}{\eta }\lambda \sqrt{\pi } \left( \rm{Erf}\left[ \lambda C_{\zeta }\left( t \right) \right] - \rm{Erf}\left[ \lambda C_{\zeta }\left( 0 \right) \right] \right) } \,{} $$

with $\rm{Erf}\left( z\right)$ indicating the Gauss error function,

$$ \begin{aligned} & \lambda \equiv \sqrt{\frac{\kappa _\mathrm{bin}}{2 \alpha _\mathrm{syn}}} \,\text {,} \quad C_{\zeta }\left( t \right) \equiv - \rm{N}_\mathrm{b}^\mathrm{max} \\ &\quad+ \rm{N}_\mathrm{b}(0) + \rm{N}_\mathrm{u}(0) + \alpha _\mathrm{syn}t \,\text {,} \quad {\eta }\equiv \left( 1 - \frac{\rm{N}_\mathrm{b}(0)}{\rm{N}_\mathrm{b}^\mathrm{max}}\right) \,\text {,}{} \end{aligned} $$

and all further symbols defined in the main text.

### Experimental methods

The experimental setup was previously described in [26]. For visualization of on-chip dynamic expression, Fig. 8, the chip was placed in an incubator chamber (Bold-line stage top incubator, Okolab) installed on the microscope and set to 30 ${}^{\circ }\text {C}$. Fluorescent images were obtained using an AxioObserver Z1 inverted microscope with a motorized stage (Zeiss), 10x/0.3 MPlanFL N (Olympus) Objective. Illumination was performed using a Colibri2 LED illumination system equipped with a 470 nm LED module (Zeiss) and filter sets 38 HE (Zeiss, excitation 470/40  nm, dichroic mirror 495 nm, emission 525/50  nm). Images were captured using an iXon Ultra CCD camera (Andor Technology, Belfast, UK). Chip alignment and multi-image time series acquisition was performed using the Zeiss ZEN 2012 software. Images were analyzed as previously described in [26].

### Supplementary Information

**Additional file 1:** Additional parameters and trap distributions.

**Additional file 2: Movie S1.** Dynamics of GFP expression, diffusion and binding for different trap patterns. Montage of three wells, each with different trap pattern density, as explained in Fig. 8. Expression originates from immobilized DNA at ~150 µm from the left side of the well. Scale bar 100 µm. Images were acquired every 3 minutes.

## Data Availability

All data generated or analyzed during this study are included in this published article and its supplementary information files.
