# Supplementary material for: Computational analysis of protein synthesis, diffusion, and binding in compartmental biochips
Source: Microb Cell Fact. 2023 Nov 30;22:244. doi: 10.1186/s12934-023-02237-5 (PMC10688499; doi:10.1186/s12934-023-02237-5)
Supplement: Supplementary file 1 — Additional file 1: Additional parameters and trap distributions. [file 12934_2023_2237_MOESM1_ESM.pdf]

Supplemental Information

Computational analysis of protein synthesis, diffusion, and binding in compartmental biochips

Stefanie Förste[1], Ohad Vonshak[2], Shirley S. Daube[2], Roy H. Bar-Ziv[2], Reinhard Lipowsky[1], and Sophia Rudorf[3,4]

[1]Theory and Bio-Systems, Max Planck Institute of Colloids and Interfaces, 14476 Potsdam, Germany
[2]Department of Chemical and Biological Physics, The Weizmann Institute of Science, Rehovot 7610001, Israel
[3]Institute of Cell Biology and Biophysics, Leibniz University Hannover, 30419 Hannover, Germany
[4]corresponding author

1 Parameters

The following parameter sets were used to generate the figures of the main text:

Table 1: **Parameters for Figs. 2 b) and 2 c).**
$C_b^{max} = 1\,\mu M$, $C_b(0) = C_u(0) = 0\,\mu M$.

α_{syn} $[\mu M\,s^{-1}]$	κ_{bin} $[s^{-1}\,\mu M^{-1}]$
1e-05	1e-05
1e-05	2e-05
1e-05	4e-05
1e-05	0.0001
1e-05	0.0002
1e-05	0.0004
1e-05	0.001
1e-05	0.002
1e-05	0.004
1e-05	0.01
1e-05	0.02
1e-05	0.04
1e-05	0.1
1e-05	0.2
1e-05	0.4
2e-05	1e-05
2e-05	2e-05
2e-05	4e-05
2e-05	0.0001
2e-05	0.0002
2e-05	0.0004
2e-05	0.001
2e-05	0.002
2e-05	0.004
2e-05	0.01
2e-05	0.02
2e-05	0.04

Table 1: Parameters for Figs. 2 b) and 2 c) continued.

α_{syn} $\left[\mu\text{M}\,\text{s}^{-1}\right]$	κ_{bin} $\left[\text{s}^{-1}\,\mu\text{M}^{-1}\right]$
2e-05	0.1
2e-05	0.2
2e-05	0.4
4e-05	1e-05
4e-05	2e-05
4e-05	4e-05
4e-05	0.0001
4e-05	0.0002
4e-05	0.0004
4e-05	0.001
4e-05	0.002
4e-05	0.004
4e-05	0.01
4e-05	0.02
4e-05	0.04
4e-05	0.1
4e-05	0.2
4e-05	0.4
0.0001	1e-05
0.0001	2e-05
0.0001	4e-05
0.0001	0.0001
0.0001	0.0002
0.0001	0.0004
0.0001	0.001
0.0001	0.002
0.0001	0.004
0.0001	0.01
0.0001	0.02
0.0001	0.04
0.0001	0.1
0.0001	0.2
0.0001	0.4
0.0002	1e-05
0.0002	2e-05
0.0002	4e-05
0.0002	0.0001
0.0002	0.0002
0.0002	0.0004
0.0002	0.001
0.0002	0.002
0.0002	0.004
0.0002	0.01
0.0002	0.02
0.0002	0.04
0.0002	0.1
0.0002	0.2
0.0002	0.4
0.0004	1e-05
0.0004	2e-05
0.0004	4e-05
0.0004	0.0001
0.0004	0.0002
0.0004	0.0004
0.0004	0.001
0.0004	0.002

Table 1: Parameters for Figs. 2 b) and 2 c) continued.

α_{syn} $[\mu\text{M}\,\text{s}^{-1}]$	κ_{bin} $[\text{s}^{-1}\,\mu\text{M}^{-1}]$
0.0004	0.004
0.0004	0.01
0.0004	0.02
0.0004	0.04
0.0004	0.1
0.0004	0.2
0.0004	0.4
0.001	1e-05
0.001	2e-05
0.001	4e-05
0.001	0.0001
0.001	0.0002
0.001	0.0004
0.001	0.001
0.001	0.002
0.001	0.004
0.001	0.01
0.001	0.02
0.001	0.04
0.001	0.1
0.001	0.2
0.001	0.4
0.002	1e-05
0.002	2e-05
0.002	4e-05
0.002	0.0001
0.002	0.0002
0.002	0.0004
0.002	0.001
0.002	0.002
0.002	0.004
0.002	0.01
0.002	0.02
0.002	0.04
0.002	0.1
0.002	0.2
0.002	0.4
0.004	1e-05
0.004	2e-05
0.004	4e-05
0.004	0.0001
0.004	0.0002
0.004	0.0004
0.004	0.001
0.004	0.002
0.004	0.004
0.004	0.01
0.004	0.02
0.004	0.04
0.004	0.1
0.004	0.2
0.004	0.4
0.01	1e-05
0.01	2e-05
0.01	4e-05
0.01	0.0001

Table 1: Parameters for Figs. 2 b) and 2 c) continued.

α_{syn} $\left[\mu\mathrm{M\,s^{-1}}\right]$	κ_{bin} $\left[\mathrm{s^{-1}\,\mu M^{-1}}\right]$
0.01	0.0002
0.01	0.0004
0.01	0.001
0.01	0.002
0.01	0.004
0.01	0.01
0.01	0.02
0.01	0.04
0.01	0.1
0.01	0.2
0.01	0.4
0.02	1e-05
0.02	2e-05
0.02	4e-05
0.02	0.0001
0.02	0.0002
0.02	0.0004
0.02	0.001
0.02	0.002
0.02	0.004
0.02	0.01
0.02	0.02
0.02	0.04
0.02	0.1
0.02	0.2
0.02	0.4
0.04	1e-05
0.04	2e-05
0.04	4e-05
0.04	0.0001
0.04	0.0002
0.04	0.0004
0.04	0.001
0.04	0.002
0.04	0.004
0.04	0.01
0.04	0.02
0.04	0.04
0.04	0.1
0.04	0.2
0.04	0.4
0.1	1e-05
0.1	2e-05
0.1	4e-05
0.1	0.0001
0.1	0.0002
0.1	0.0004
0.1	0.001
0.1	0.002
0.1	0.004
0.1	0.01
0.1	0.02
0.1	0.04
0.1	0.1
0.1	0.2
0.1	0.4

Table 1: Parameters for Figs. 2 b) and 2 c) continued.

α_{syn} $\left[\mu\text{M}\,\text{s}^{-1}\right]$	κ_{bin} $\left[\text{s}^{-1}\,\mu\text{M}^{-1}\right]$
0.2	1e-05
0.2	2e-05
0.2	4e-05
0.2	0.0001
0.2	0.0002
0.2	0.0004
0.2	0.001
0.2	0.002
0.2	0.004
0.2	0.01
0.2	0.02
0.2	0.04
0.2	0.1
0.2	0.2
0.2	0.4
0.4	1e-05
0.4	2e-05
0.4	4e-05
0.4	0.0001
0.4	0.0002
0.4	0.0004
0.4	0.001
0.4	0.002
0.4	0.004
0.4	0.01
0.4	0.02
0.4	0.04
0.4	0.1
0.4	0.2
0.4	0.4

Table 2: **Parameters for Figs. 2 d) and 2 e).**
$\text{C}_\text{b}^\text{max} = 1\,\mu\text{M}$, $\text{C}_\text{b}(0) = \text{C}_\text{u}(0) = 0\,\mu\text{M}$.

α_{syn} $\left[\mu\text{M}\,\text{s}^{-1}\right]$	κ_{bin} $\left[\text{s}^{-1}\,\mu\text{M}^{-1}\right]$
0.001	0.001
0.001	0.1
0.1	0.1

Table 3: **Parameters for Fig. 3.**
$\text{C}_\text{b}^\text{max} = 1\,\mu\text{M}$, $\text{C}_\text{b}(0) = \text{C}_\text{u}(0) = 0\,\mu\text{M}$.

α_{syn} $\left[\mu\text{M}\,\text{s}^{-1}\right]$	κ_{bin} $\left[\text{s}^{-1}\,\mu\text{M}^{-1}\right]$	$\widetilde{D}$ $\left[\text{s}^{-1}\right]$
0.001	0.1	0.001
0.1	0.1	0.001
0.001	0.1	0.1
0.1	0.1	0.1

Table 4: **Parameters for Fig. 4.**
$C_b^{max} = 1\,\mu M$, $C_u(0) = C_b(0) = 0\,\mu M$, synthesis in boxes $i_{syn} = 12, \ldots 19$.

$\alpha_{syn}\Delta t$ [µM]	$\kappa_{bin}\Delta t$ [µM^{-1}]	$\widetilde{D}\Delta t$
0.0002	0.0002, 0.002, 0.02	0.08
0.0002	0.02	0.08, 0.03, 0.01
0.0001, 0.0002, 0.0003	0.02	0.08

Table 5: **Parameters for Fig. 5.**
$C_b^{max} = 1\,\mu M$, $C_u(0) = C_b(0) = 0\,\mu M$, synthesis in boxes $i_{syn} = 12, \ldots 19$. Trap distribution as shown in Fig. S1#14.

$\alpha_{syn}\Delta t$ [µM]	$\kappa_{bin}\Delta t$ [µM^{-1}]	$\widetilde{D}\Delta t$
0.0002	0.0002	0.083

Table 6: **Parameters for Figs. 6 b) and 6 c).**
$C_b^{max} = 1\,\mu M$, $C_u(0) = C_b(0) = 0\,\mu M$, synthesis in boxes $i_{syn} = 7, \ldots 14$.

$\alpha_{syn}\Delta t$ [µM]	$\kappa_{bin}\Delta t$ [µM^{-1}]	$\widetilde{D}\Delta t$	trap distribution as shown in Fig. S1
0.0002	0.002	0.0098	23, 27, 31, 32, 33

Table 7: **Parameters for Fig. 7.**
$C_b^{max} = 1\,\mu M$, $C_u(0) = C_b(0) = 0\,\mu M$, synthesis in boxes $i_{syn} = 7, \ldots 14$.

$\alpha_{syn}\Delta t$ [µM]	$\kappa_{bin}\Delta t$ [µM^{-1}]	$\widetilde{D}\Delta t$
0.0002, 0.00075	0.002	0.045

Table 8: **Parameters for Fig. 8.**
$C_u(0) = C_b(0) = 0\,\mu M$, synthesis in boxes $i_{syn} = 12, \ldots 19$.

	$\alpha_{syn}\Delta t$ [µM]	$\kappa_{bin}\Delta t$ [µM^{-1}]	$\widetilde{D}\Delta t$	C_b^{max} [µM]	trap distribution as shown in Fig. S1
b	0.0002	0.002	0.03	1	39
d	0.0002	0.002	0.03	1	10
f	0.0002	0.002	0.03	2	11

2 Trap distributions

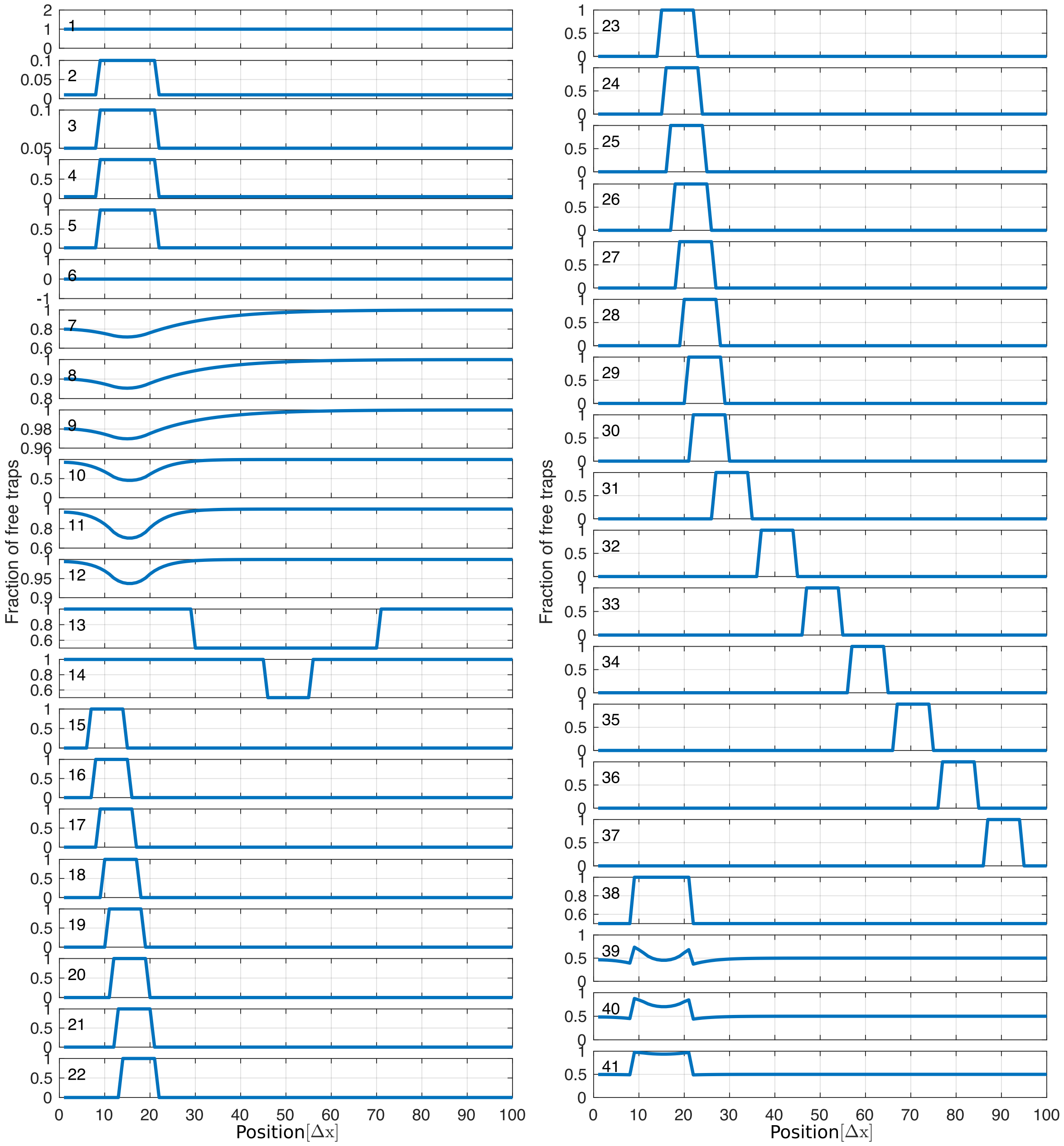

Figure 1: **Trap distributions.** At the beginning of a simulation of a 100-box model, an initial concentration of free traps is assumed (blue lines), which can be either homogeneously or heterogeneously distributed. Distributions # 7 to 12 and 39 to 41 are motivated by the experimental setup and imitate slight inhomogeneities in the free trap coverage of the biochip in proximity to the DNA brushes.
